# Spatial Dynamic Functional Connectivity Analysis Identifies Distinctive Biomarkers in Schizophrenia

**DOI:** 10.3389/fnins.2019.01006

**Published:** 2019-09-24

**Authors:** Suchita Bhinge, Qunfang Long, Vince D. Calhoun, Tülay Adali

**Affiliations:** ^1^Department of Computer Science and Electrical Engineering, University of Maryland Baltimore County, Baltimore, MD, United States; ^2^The Mind Research Network, Albuquerque, NM, United States; ^3^Department of Electrical and Computer Engineering, University of New Mexico, Albuquerque, NM, United States

**Keywords:** prediction, dynamic functional connectivity, independent vector analysis, schizophrenia, spatio-temporal, states

## Abstract

Dynamic functional network connectivity (dFNC) analysis is a widely-used to study associations between dynamic functional correlations and cognitive abilities. Traditional methods analyze time-varying association of different spatial networks while assuming that the spatial network itself is stationary. However, there has been very little work focused on voxelwise spatial variability. Exploiting the variability across both the temporal and spatial domains provide a more promising direction to obtain reliable dynamic functional patterns. However, methods for extracting time-varying spatio-temporal patterns from large-scale functional magnetic resonance imaging (fMRI) data present some challenges, such as degradation in performance with respect to increase in size of the data, estimation of the number of dynamic components, and the potential sensitivity of the resulting dFNCs to selection of the networks. In this work, we implement subsequent extraction of exemplars and dynamics using a constrained independent vector analysis, a data-driven method that efficiently estimates spatial and temporal dynamics from large-scale resting-state fMRI data. We explore the benefits of analyzing spatial dFNC (sdFNC) patterns over temporal dFNC (tdFNC) patterns in the context of differentiating healthy controls and patients with schizophrenia. Our results indicate that for resting-state fMRI data, sdFNC patterns were able to better classify patients and controls, and yield more distinguishing features compared with tdFNC patterns. We also estimate structured patterns of connectivity/states using sdFNC patterns, an area that has not been studied so far, and observe that sdFNC was able to successfully capture distinct information from healthy controls and patients with schizophrenia. In addition, sdFNC patterns were also able to identify functional patterns that associate with signs of paranoia and abnormalities in the patients group. We also observe that patients with schizophrenia tend to switch to or stay in a state corresponding to a hyperconnected brain network.

## 1. Introduction

Dynamic functional network connectivity (dFNC) analyzes the time-varying associations among different regions of the brain and has been widely studied in order to identify correlations between functional changes and cognitive abilities (Fox et al., 2005; Chang and Glover, 2010; Sakoğlu et al., 2010). In order to identify these functional patterns of different brain regions, conventional methods identify groups of temporally coherent voxels, referred to as spatial maps, and their corresponding activation patterns, referred to as time courses (Lee et al., 2013). Followed by the estimation of time courses, a sliding window is applied on the time courses that divides it into consecutive windows and an analysis on the time points within each window is performed (Allen et al., 2014). The analysis of dFNC patterns depends on the length of the window, where the use of a longer window length increases the risk of averaging the temporal fluctuations of interest resulting in false negatives (Preti et al., 2017), and the use of a shorter window length has too few samples for a reliable computation of correlation (Hero and Rajaratnam, 2016), resulting in the temporal variations to capture spurious fluctuations and increasing the risk of false positives (Sakoğlu et al., 2010; Hutchison et al., 2013; Leonardi and Van De Ville, 2015). Previous studies have shown that a window length between 30 and 60 s successfully estimates temporal fluctuations in resting-state functional magnetic resonance imaging (fMRI) data (Preti et al., 2017), and for most cases higher window lengths do not alter the results significantly (Keilholz et al., 2013; Li et al., 2014; Liégeois et al., 2016). However, there is a lower bound in being able to capture fluctuations due to the limited number of samples, limiting the use of dFNC analysis in the temporal domain.

Conventional methods also estimate the time-varying FNC patterns of the spatial networks while assuming that the spatial network itself is stationary. However, studies have shown that changes in the FNC patterns imply changes in the spatial networks (Calhoun et al., 2008). Hence, spatio-temporal dFNC analysis relaxes the assumption of stationarity in both the spatial and temporal domain, and provides a more general framework for capturing time-varying FNC patterns (Ma et al., 2014; Kottaram et al., 2018; Kunert-Graf et al., 2018). The availability of higher number of samples in the spatial domain also guarantees reliable estimation of functional correlations (Hero and Rajaratnam, 2016), thus providing a promising direction for the use of spatial domain for dFNC analysis. However, the methods used to extract time-varying spatio-temporal patterns face few challenges. Region of interest based methods use pre-defined resting-state networks causing the estimated functional connectivity to be sensitive to network selection. Dynamic mode decomposition, a spatio-temporal modal decomposition technique, requires significant dimension reduction that may restrict the method to estimate fewer dynamic components (Kunert-Graf et al., 2018). Independent vector analysis (IVA) provides a general and flexible framework to spatio-temporal dFNC analysis and estimates window-specific time courses and spatial maps. However its performance degrades with increase in the size of the data, for a given number of samples (Bhinge et al., 2019). Hence, in this work, we use a data-driven method to jointly extract spatio-temporal patterns using the subsequent extraction of exemplar and dynamic components using constrained IVA (SED-cIVA) method (Bhinge et al., 2019), from a large-scale fMRI data acquired from 91 healthy controls and 88 patients with schizophrenia. This two-stage method preserves variability in both domains while addressing the issue with large-scale data, by using a parameter tuning technique. This parameter tuning technique adapts to the variability of each brain region separately, thus allowing accurate estimation of time-varying spatial maps and corresponding time courses.

Although exploiting variability in the spatial domain for dFNC analysis has shown better performance in terms of classification using a seed-based analysis (Kottaram et al., 2018), which is sensitive to the networks selected, the features extracted from sdFNC patterns are not explored. In this work, we explore the use of spatial domain for dFNC analysis in order to demonstrate the benefits of exploiting variability in the spatial domain and taking advantage of the large sample size in this domain, using a data-driven approach. We perform a prediction technique to compare the ability of temporal dFNC (tdFNC) patterns and sdFNC patterns to predict if a subject is a patient or a control. We also perform a joint analysis by combining the sdFNC and tdFNC patterns together in order to explore the contribution of each toward prediction and observe that the use of sdFNC patterns alone provides higher prediction accuracy than using tdFNC patterns, or a combined feature set. This shows that exploiting the variability and taking advantage of large sample size in the spatial domain provides meaningful discriminative features. We also obtain structured patterns of connectivity/states from sdFNC patterns and identify differences between patients and controls in terms of dwell time, transition matrix and fraction of time spent in each state. To the best of our knowledge, no study has been performed to identify these properties from sdFNC patterns. Our results indicate that patients tend to stay in or transition between states associated with hyperconnected brain network. We also find significant associations between the resulting functional connectivity and signs of paranoia in the patient group using sdFNC patterns.

The remainder of the paper is organized as follows. Section 2 introduces the dataset used in this work and the method used for extraction of spatio-temporal dynamic patterns. This section also discusses the prediction technique followed by the method used for estimating states. Section 3 shows the results and discusses these results. Section 4 concludes the paper.

## 2. Materials and Methods

### 2.1. Material

We work with resting-state fMRI data is acquired from *K* = 179 subjects including 91 healthy controls (HCs) (average age: 38 ± 12) and 88 patients with schizophrenia (SZs) (average age: 37 ± 14). The dataset was obtained from the Center for Biomedical Research Excellence (COBRE) (Aine et al., 2017) and is available on the collaborative informatics and neuroimaging suite data exchange repository (https://coins.trendscenter.org/) (Scott et al., 2011). The data was obtained over the duration of 5 min with a sampling period of 2 s yielding 150 timepoints per subject. The subjects were asked to keep their eyes open during the entire scanning period. Each subject's data was pre-processed to account for motion correction, slice-time correction, spatial normalization and was slightly re-sampled to 3 × 3 × 3mm^3^ yielding 53 × 63 × 46 voxels. The first 6 time points were removed to account for the T1-effect. We perform masking on each image volume to remove the non-brain voxels and flatten the result to form an observation vector of *V* = 58,604 voxels, giving *T* = 144 time evolving observations for each subject.

### 2.2. SED-cIVA

**Figure 1:**
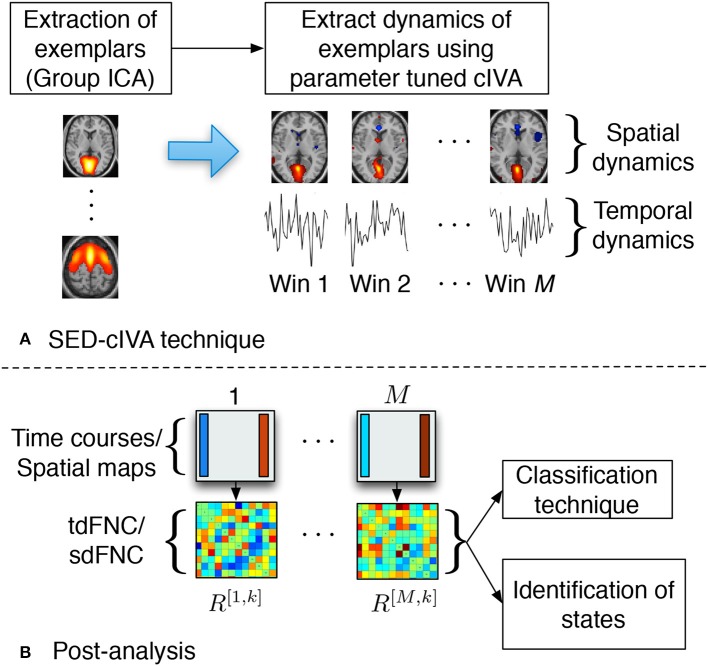
**(A)** SED-cIVA technique consists of two stages. In the first stage exemplars of resting-state networks are extracted using GICA. In the second stage, these exemplars are used as reference signals in a sliding-window cIVA framework to extract their spatial and temporal dynamics. **(B)** Spatial or temporal dFNC matrices are obtained at each time window by computing the Pearson's correlation coefficient between each pair of spatial maps or time course, respectively. These matrices are further used to classify subject as a patient or a control, and also to identify states.

In this work, we use the SED-cIVA method to extract time-varying spatial and temporal patterns. SED-cIVA consists of two stages: the first stage extracts exemplar/informative components from all subjects and uses these components as reference signals in a sliding-window parameter-tuned cIVA framework in the second stage, to obtain the time-varying representation of these components. The idea of SED-cIVA is to extract stationary representation of the most informative resting-state networks from the given dataset, in the first stage followed by estimating the time-varying representation of these networks for each subject using a sliding-window approach. Figure 1A shows the flow-chart of the method.

#### 2.2.1. Extraction of Exemplars

SED-cIVA provides flexibility in the choice of the method used to extract the exemplar components. Templates of resting-state networks of interest that are pre-defined based on extensive studies of resting-state fMRI data can be used as exemplars. Sparsity-learning methods such as dictionary learning (Varoquaux et al., 2011) or sparse ICA (Boukouvalas et al., 2017) can be used to extract more focal spatial components. One of the widely used methods for extraction of components from multiple subjects is group independent component analysis (GICA) that estimates a common subspace consisting of the most informative components across all subjects (Calhoun et al., 2001a,b). In this work, we perform GICA on all subjects to extract exemplars of resting-state networks using the group ICA for fMRI toolbox (GIFT). GICA performs a subject-level principal component analysis (PCA) to extract the signal subspace for each subject followed by a group-level PCA on the principal components (PCs) from all the subjects. In order to exploit higher order statistics, it performs independent component analysis (ICA) on the group-level PCs. We estimate the model order for each subject using the minimum description length criterion that accounts for sample dependence (Li et al., 2007) and the final order is selected as the mean (30) plus one standard deviation (5) across all subject's model orders. The dimension of the signal subspace in the subject-level PCA stage is set as 53 and the order for the group-level PCA stage is set as 35. By default, GIFT selects the subject-level PCA order (53) to be 1.5 times the final order (35). We use the entropy rate bound minimization (ERBM) algorithm (Li and Adali, 2010) to estimate the group-level independent components. ERBM is a flexible ICA algorithm that exploits multiple statistical properties of the sources such as sample dependence and higher order statistics, and provides a better estimation of fMRI sources (Long et al., 2018a). The ICA algorithm is run 10 times and the best run is selected using the minimum spanning tree approach (Du et al., 2014). Among the 35 group-level components, we visually select *N* = 17 components as exemplars, denoted as **d**_*n*_, *n* = 1, …, 17, and these components are used as reference signals in the second stage.

#### 2.2.2. Extraction of Spatial and Temporal Dynamics of Exemplars

In the second stage, time-varying representation of the exemplar components is estimated using a sliding-window IVA approach. In the sliding-window approach, each subject's data is divided into *M* = 17 windows of length *L* = 16 (32 s) with a 50% overlap. We extract time-varying spatio-temporal patterns of the exemplars by performing parameter-tuned cIVA on each subject's data. Parameter-tuned cIVA is a type of IVA that incorporates information regarding the exemplars into the IVA framework and extracts window-specific time courses and spatial maps of these exemplars. The general IVA model, for a given set of observations, can be written as **X**^[*m*]^ = **A**^[*m*]^**S**^[*m*]^, *m* = 1, …, *M*, where **X**^[*m*]^ ∈ ℝ^*L*×*V*^ denotes the observations from window *m*, **A**^[*m*]^ ∈ ℝ^*L*×*L*^ denotes the mixing matrix and the rows in **S**^[*m*]^ ∈ ℝ^*L*×*V*^ are the latent sources. IVA estimates *M* demixing matrices, **W**^[*m*]^, such that the sources within each dataset are maximally independent and sources across dataset are maximally dependent. The cost function, written using random vector notation, is given as (Anderson et al., 2012; Adali et al., 2014),

(1) $$\begin{array}{l} J_{\text{IVA}}=\overset{L}{\underset{l=1}{\sum }}\left[\overset{M}{\underset{m=1}{\sum }}H\left({\hat{s}}_{l}^{\left[m\right]}\right)-I\left({\hat{\text{s}}}_{l}\right)\right]-\overset{M}{\underset{m=1}{\sum }}\log|\det{\text{W}}^{\left[m\right]}|, \end{array}$$

where $H\left({ŝ}_{l}^{\left[m\right]}\right)$ denotes the entropy of the *l*th source estimate for the *m*th dataset, and $I\left({\text{s}}_{l}\right)$ denotes the mutual information of the *l*th source component vector (SCV) estimate, ${\hat{\text{s}}}_{l}^{\text{T}}=\left[{ŝ}_{l}^{\left[1\right]},\ldots ,{ŝ}_{l}^{\left[M\right]}\right]$. The optimization of Equation (1) jointly weighs the independence within the dataset through the entropy term along with the log determinant term and dependence across the datasets through the mutual information term. The dependent sources across datasets can be grouped together to form a SCV. Thus, in the SED-cIVA framework, IVA treats each window as a dataset and estimates window-specific time courses and spatial maps, and a SCV represents the time-varying representation of a spatial map. Parameter-tuned cIVA directs the estimation of the sources toward the reference components through an additional term in the IVA cost function given as,

(2) $$\begin{array}{l} J=J_{\text{IVA}}-\overset{L}{\underset{l=1}{\sum }}\frac{1}{2\gamma_{n}}\overset{M}{\underset{m=1}{\sum }}\left\{{\left[\max\left\{0,\mu_{n}^{\left[m\right]}+\gamma_{n}g\left({\hat{\text{s}}}_{n}^{\left[m\right]},{\text{d}}_{n}\right)\right\}\right]}^{2}-{\left(\mu_{n}^{\left[m\right]}\right)}^{2}\right\}, \end{array}$$

where $\mu_{n}^{\left[m\right]}$ is the regularization parameter, γ_*n*_ is a positive scalar and $g\left({\hat{\text{s}}}_{l}^{\left[m\right]},{\text{d}}_{n}\right)$ is the inequality constraint function given as,

(3) $$\begin{array}{l} g\left({\hat{\text{s}}}_{l}^{\left[m\right]},{\text{d}}_{n}\right)=\rho_{n}-\left|\text{corr}\left({\hat{\text{s}}}_{l}^{\left[m\right]},{\text{d}}_{n}\right)\right|\leq 0. \end{array}$$

The constraint parameter, ρ_*n*_, controls the amount of correspondence between the exemplar, **d**_*n*_, and the estimated source, and acts as a lower bound for the amount of correlation between them. A higher value for this parameter enforces the estimated source to be exactly similar to the exemplar component, not allowing the other components to interact with the exemplar component. On the other hand, a lower value deviates the estimated source from the exemplar component causing the source to be prone to noise and other artifacts. Additionally, the interaction between an exemplar component and other components vary with respect windows and subjects, hence a fixed value for this parameter across all *m* and for all subjects does not allow the model to efficiently capture the variability across windows. Thus in order to capture the variability, SED-cIVA implements parameter-tuned cIVA, that adaptively selects a value from a set of possible values for ρ_*n*_, denoted as P, as follows,

(4) $$\begin{array}{l} {\hat{\rho }}_{n}=\arg\underset{\rho_{n}\in P}{\min}\left[\underset{m}{\min}{\left\{\left|\rho_{n}-|\text{corr}\left({\hat{\text{s}}}_{l}^{\left[m\right]},{\text{d}}_{n}\right)|\right|\right\}}_{m=1}^{M}\right],\\ \;P\in \left\{0.001,0.1,\ldots \;,0.9\right\} \end{array}$$

This updates computes $g\left({\hat{\text{s}}}_{l}^{\left[m\right]},{\text{d}}_{n}\right)$ for all *m* and for each value in set P and selects the highest value of ρ_*n*_ from set P that satisfies the condition in Equation (3) for all windows. From Equation (3), we observe that $\rho_{n}\leq \left|\text{corr}\left({\hat{\text{s}}}_{l}^{\left[m\right]},{\text{d}}_{n}\right)\right|$, allowing ρ_*n*_ to be between 0 and 1. Thus, we define the set P as the possible values between 0 and 1. Hence, parameter-tuned cIVA selects the highest lower bound using Equation (4) and adaptively tunes itself with respect to each exemplar component, allowing the method to capture variability across windows. The use of exemplars also guides the solution toward the optimal solution addressing the issue of large-scale data that is observed in regular IVA, and relaxes the independence assumption of IVA (Bhinge et al., 2017). Hence SED-cIVA effectively captures variability of the exemplars across windows and subjects.

In parameter-tuned cIVA, we constrain one source at a time, ${\hat{\text{s}}}_{1}^{\left[m\right]}$, to one of the exemplar components, **d**_*n*_, whereas the rest of the sources, ${\hat{\text{s}}}_{l}^{\left[m\right]},l=2,\ldots ,L$, are unconstrained. For each **d**_*n*_, we obtain 10 solutions using parameter-tuned cIVA with γ_*n*_ = 3, *n* = 1, …, *N*, using the IVA-L-SOS algorithm for different random initializations and select the most consistent run using the method described in Long et al. (2018b). IVA-L-SOS is a type of IVA algorithm that assumes the sources are multivariate Laplacian distributed and exploits second order statistics (SOS) (Bhinge et al., 2019). This algorithm provides a better match to the properties of fMRI sources, since fMRI sources are in general expected to have a super-Gaussian distribution, like Laplacian (Calhoun and Adali, 2012), and are correlated across windows. The estimated sources corresponding to the constrained exemplars from the consistent run are denoted as ${\text{y}}_{n}^{\left[m,k\right]},n=1,\ldots ,N,m=1,\ldots ,M,k=1,\ldots ,K$, whereas the corresponding time courses are denoted as ${\text{a}}_{n}^{\left[m,k\right]}$.

### 2.3. Post-processing

SED-cIVA estimates time-varying spatial components and corresponding time courses for each exemplars that are further used to compute spatial and temporal dFNC matrices. The time courses at each window are further processed to remove quadratic, linear and cubic trends, and low-pass filtered with a cutoff frequency of 0.15 Hz (Allen et al., 2014). The tdFNC matrix at the *m*th time window for the *k*th subject is denoted as *R*^[*m,k*]^. Each element in *R*^[*m,k*]^ is obtained by computing the Pearson's correlation coefficient between each pair of the time courses in that time window, $r_{ij}^{\left[m,k\right]}=\text{corr}\left({\text{a}}_{i}^{\left[m,k\right]},{\text{a}}_{j}^{\left[m,k\right]}\right),i,j=1,\ldots ,N$. Similarly sdFNC matrices are obtained by computing the Pearson's correlation coefficient between each pair of spatial maps at each time window. The correlation can be interpreted as the similarity in the activated sources in the spatial maps. Although IVA, like ICA, estimates spatial maps that are maximally independent within a time window, it also groups together the voxels that have a similar activation pattern (Calhoun and de Lacy, 2017). Hence, if in a time window two sources have a similar activation pattern these sources would be estimated as a single spatial map. Hence, we would expect a high correlation between the estimated spatial maps of the corresponding constrained sources. These matrices are further used to classify subjects as patients or controls, and to obtain spatial and temporal FC states. The post-analysis steps are shown in Figure 1B.

### 2.4. Prediction Technique

**Figure 2:**
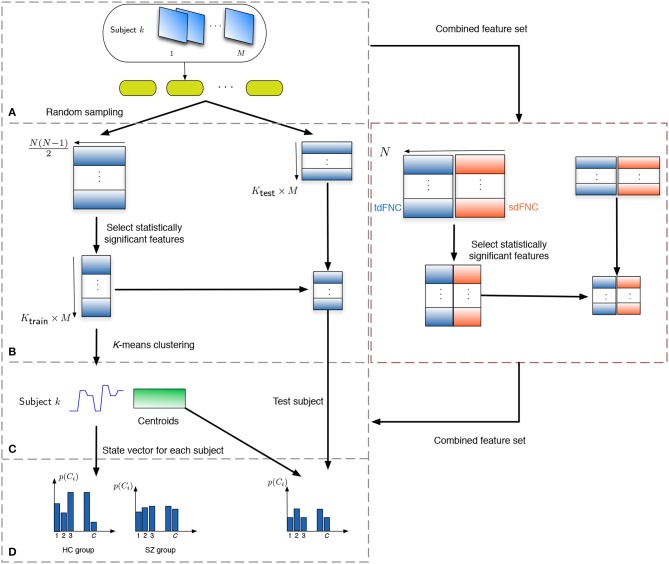
Flowchart to obtain the features for prediction. **(A)** The subjects are divided into training and testing set, where the training set consists of 150 subjects, 75 from the HC and SZ group each. The remaining 29 subjects form the testing set. **(B)** Each tdFNC/sdFNC matrix is flattened to a row and the distinguishing features are extracted using a two-sample *t*-test. The indices of the distinguishing features are recorded and used to select the corresponding features in the testing stage. In the combined feature set for joint analysis, the flattened features from both domains are concatenated in the feature dimension and similar steps are performed. **(C)** The selected features from the training set are clustered into *C* clusters using *K*-means clustering to obtain the centroids and the state vector for each subject. **(D)** The probability of occurrence of each state is computed for the HC and SZ group separately. For the testing stage the state vector for each subject is obtained using the centroids from the training stage and probability of occurrence for each state is computed.

In order to study how informative the spatial and temporal dFNC features are, we evaluate the predictability of these features in terms of predicting if a subject is a patient or a control. Note that the aim of this experiment is to observe potential advantages of sdFNC features and not the actual prediction accuracy, hence we use a simpler Naïve Bayes classifier that does not require tuning of parameters. The flowchart for the prediction technique is shown in Figure 2. We obtain 1,000 independent Monte-Carlo subsamplings of the data. In each subsampling, subjects from HC and SZ group are divided into training and testing sets, where each training group consists of 75 randomly sampled subjects from the HC and the SZ group (*K*_train_ = 150). The remaining subjects form the testing set (*K*_test_ = 29). We then obtain *K*_train_ × *M* observations of *N*(*N* − 1)/2 dimensional features from the tdFNC/sdFNC matrices. In order to select the distinguishing features from the *N*(*N* − 1)/2 features, we perform a two-sample *t*-test on the features from the HC and SZ group as shown in Figure 2B. Features that demonstrate significant difference (*p* < 0.05) are used in further stages. The indices of the significant features are recorded and used in the testing stage. This feature selection is done separately for tdFNC and sdFNC matrices. The selected features are clustered into *C* clusters, where in this experiment we vary the number of clusters from 3 to 30. For training the Naïve Bayes classifier, we obtain the probability of each state for the HC group and SZ group, $p^{g}\left(C_{i}\right),g=\left\{\text{HC, SZ}\right\}$. In the testing stage, the features that indicated significant difference in the training stage were selected and each observation from a test subject is assigned a state with maximum Pearson's correlation between the observation and the cluster centroid. We then obtain the probability of each state using $p^{\left[k\right]}\left(C_{i}\right)=n_{i}^{\left[k\right]}/M,i=1,\ldots ,C$ and use the test feature vector to predict the class of the subject. A test subject is assigned to HC or SZ group using the following rule,

(5) $$\begin{array}{l} ŷ=\arg\underset{g=\left\{\text{HC},\text{SZ}\right\}}{\max}p\left(g\right)\overset{C}{\underset{i=1}{\prod }}{\left[p^{g}\left(C_{i}\right)\right]}^{n_{i}} \end{array}$$

where *n*_*i*_ denotes the number of occurrences of state *i* in the test subject. Steps (B–D) from Figure 2 are performed for each sub-sampling of the data.

For the joint analysis of spatio-temporal features, the sdFNC and tdFNC features selected after the two-sample *t*-test on these feature sets separately, are concatenated in the feature dimension to study the effect of combining the two feature sets on prediction accuracy. We compare the results from the combined feature set with the results from using sdFNC and tdFNC feature set alone. Table 1 provides some inferences regarding the comparison results. Let *Q*_S_ denote the prediction accuracy obtained using sdFNC matrices, *Q*_T_ denote the prediction accuracy obtained using tdFNC matrices and *Q*_ST_ denote the prediction accuracy obtained using the combined feature set. We can say that if the prediction accuracy increases after combining the sdFNC and tdFNC features, both feature sets provide unique discriminative features, whereas if the prediction accuracy using sdFNC features is greater than *Q*_ST_, then tdFNC provide non-discriminative features, hindering the classification performance.

**Table 1:** Inferences about predictability of sdFNC, tdFNC, and combined feature set.

*Q*_ST_ > *Q*_S_, *Q*_T_	sdFNC and tdFNC yield unique discriminative features jointly contributing to classify subjects
*Q*_ST_ < *Q*_S_, *Q*_T_	sdFNC and tdFNC both yield non-discriminative features that are unable to classify subjects
*Q*_S_ or *Q*_T_ > *Q*_ST_ > *Q*_T_ or *Q*_S_	tdFNC or sdFNC yield non-discriminative features affecting the prediction
*Q*_ST_ = *Q*_S_ or *Q*_T_	tdFNC or sdFNC are not providing additional information to classify subjects

*Q_S_ denotes the prediction accuracy obtained using sdFNC matrices, Q_T_ denotes the prediction accuracy obtained using tdFNC matrices and Q_ST_ denotes the prediction accuracy obtained using the combined feature set*.

### 2.5. Identification of States

Recent studies have shown that fluctuations in the brain networks in resting-state are not random but exhibit structured patterns that vary over time (Cribben et al., 2012; Allen et al., 2014; Yang et al., 2014). In this study, we obtain these structured patterns or states using sdFNC matrices. In the first step toward identifying the states, we flatten the upper diagonal part of each correlation matrix, *R*^[*m,k*]^, to obtain a feature vector of dimension *N*(*N* − 1)/2 yielding *MK* observations. For each subject, the standard deviation across the feature dimension is computed and a subset of FNC matrices are selected corresponding to the maximum standard deviation as subject exemplars. Thus the subject exemplars represent the features that are more informative, alternatively those with higher variability. Further *k*-means clustering is performed to cluster these subject exemplars into *C* clusters using Pearson's correlation coefficient as the distance measure. The centroids resulting from clustering the subject exemplars are used as initial points to cluster the entire observation set. This two-step clustering process is performed in order to obtain a robust solution. The performance of *k*-means clustering assigns a cluster or state index to each observation resulting in a state vector for each subject. The state vector thus represents the evolution of the states over time. This vector is further analyzed to obtain the transition matrix, dwell time and fraction of time spent for each state and for each subject. The transition matrix denotes the number of transitions from state *i* to state *j*, *i, j* = {1, …, *C*}, the dwell time denotes the amount of time a subject remains in a particular state, and fraction of time spent denotes the probability of occurrence of a state.

## 3. Results and Discussion

**Figure 3:**
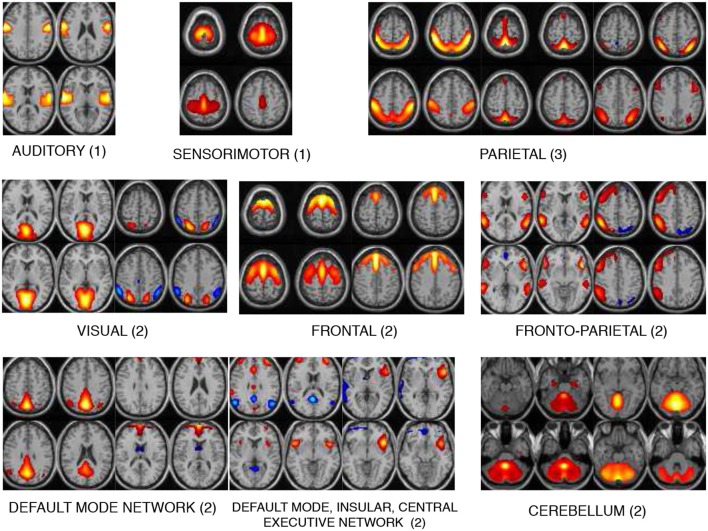
The 17 components selected are divided into 8 domains: auditory (AUD), sensorimotor (SM), frontal (FRO), fronto-parietal (FP), parietal (PAR), visual (VIS), default mode network (DMN), and cerebellum (CB). The DMN domain includes spatial maps consisting the anterior, posterior DMN, central executive network and insular (INS) components. The number indicated next to each domain name is number of components belonging to that domain.

The 17 components selected as exemplars after performing GICA are shown in Figure 3. These components are divided into 8 domains: auditory (AUD), sensorimotor (SM), frontal (FRO), fronto-parietal (FP), parietal (PAR), visual (VIS), default mode network (DMN) and cerebellum (CB). The PAR domain comprise three networks: PAR1, PAR2, and PAR3, corresponding to their peak activation located in the primary somatosensory cortex, supramarginal gyrus and somatosensory association cortex, respectively. The DMN domain consists of one component corresponding to posterior DMN, one component corresponding to anterior DMN (ADMN), one DIC network and one insular (INS) component. The DIC component shows a network of a de-activated posterior DMN component and an activated central executive network and right fronto-insular (INS) network. The VIS domain comprise two networks: VIS1 and VIS2, corresponding to their peak activation situated in the lateral and medial visual cortex, respectively. The FRO domain comprise two networks: FRO1 and FRO2 corresponding to their peak activation in the frontal cortex located anterior to the premotor cortex and dorsolateral prefrontal cortex, respectively.

### 3.1. Prediction Results

**Figure 4:**
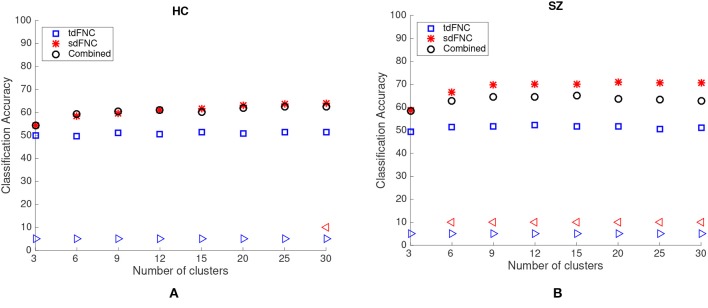
Average prediction accuracy computed over 1,000 independent Monte-Carlo samplings using tdFNC, sdFNC and combined features for **(A)** HC group and **(B)** SZ group. A blue triangle denotes significant difference between tdFNC result and combined feature set result, whereas a read triangle denotes significant difference between sdFNC result and combined feature set result. A triangle pointing left, “⊲,” indicates the prediction accuracy of tdFNC/sdFNC is greater than the combined feature set result, whereas a triangle pointing right, “⊳,” indicates the prediction accuracy of tdFNC/sdFNC is less than the combined feature set result.

The average prediction accuracies computed across 1,000 Monte Carlo subsamplings, using the sdFNC, tdFNC and combined feature set for different number of clusters is shown in Figure 4. Figure 4A shows the result for the HC group and Figure 4B shows the result for the SZ group. In order to test if the prediction accuracies computed using sdFNC and tdFNC features are significantly different from the combined feature set, we perform a permutation test using a two-sample *t*-test as the hypothesis test. The results indicate that the prediction accuracy computed using sdFNC features is significantly higher than the one computed using tdFNC and the combined feature set for the SZ group for different number of clusters. This suggests the use of tdFNC features yield non-discriminative features that degrade the prediction performance for the SZ group. For the HC group, the prediction accuracy computed using sdFNC features is higher than the one computed using tdFNC features and equal to the combined feature set for the SZ group for different number of clusters. This suggests that the tdFNC features are not providing additional information to classify subjects as controls.

We also compute the sensitivity and specificity of the prediction model obtained using sdFNC and tdFNC features. The true positives (TP) denote the percentage of SZ subjects that are correctly identified as SZ, true negatives (TN) denote the percentage of HC subjects that are correctly identified as HC, false negatives (FN) denote the percentage of SZ subjects incorrectly identified as HC, and false positives (FP) denote the percentage of HC subjects incorrectly identified as SZ. Sensitivity and specificity for each Monte Carlo subsampling is computed as follows,

$$\begin{array}{l} \text{Sensitivity}=\frac{\text{TP}}{\text{TP}+\text{FN}},\;\text{Specificity}=\frac{\text{TN}}{\text{TN}+\text{FP}}. \end{array}$$

**Figure 5:**
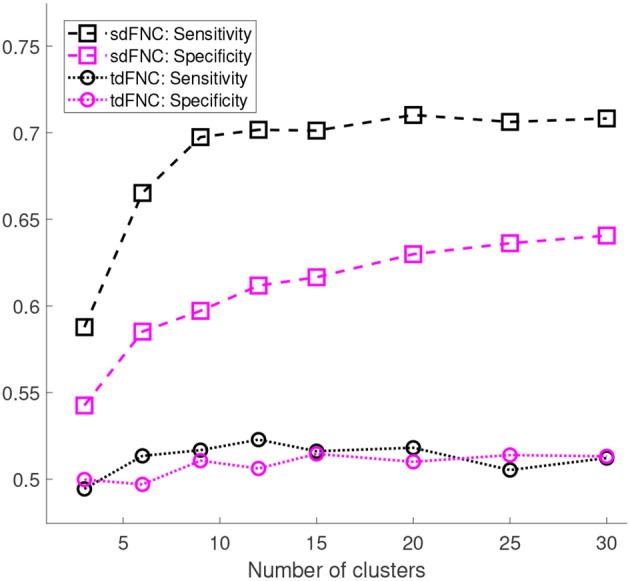
Sensitivity and specificity of the prediction model trained using sdFNC and tdFNC features. The sensitivity and specificity values are averaged over 1,000 Monte Carlo subsamplings. The results indicate that sensitivity and specificity is higher using sdFNC features compared with the tdFNC features. A higher sensitivity for sdFNC indicates a better prediction ability of these features to correctly identify SZ subjects.

Figure 5 shows the results of these measures computed for sdFNC and tdFNC features. Sensitivity and specificity values are higher using sdFNC features compared with the tdFNC features. A higher sensitivity for sdFNC features indicates that these features are better able to identify SZ subjects than HC subjects.

**Figure 6:**
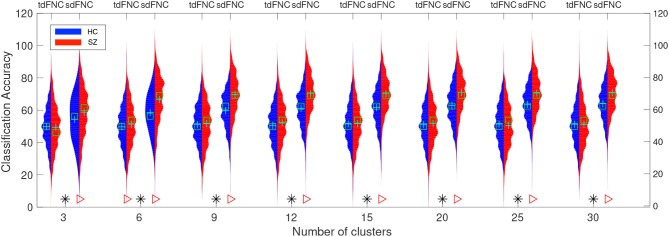
Predictability results using Naïve Bayes classifier. Red color indicates the histogram of prediction accuracies obtained for the SZ group whereas blue indicates the histogram of prediction accuracies for the HC group. X-axis denotes the number of clusters, C used to cluster the features from tdFNC/sdFNC graphs. The green “+” sign denotes the mean value and “□” sign indicates the median value. The markers at the bottom show results from a permutation test to test for statistical differences (*p* < 0.05, corrected). A “*” denotes the accuracies are significantly higher using sdFNC features compared with tdFNC features. A “⊳” denotes higher prediction accuracy for SZ group. We observe a higher prediction accuracy using sdFNC features and a significantly higher accuracy for the patients group, for different number of clusters.

In order to test for differences between the prediction accuracies using sdFNC and tdFNC features, and between the HC group and the SZ group, we perform a permutation test between these groups using a two-sample *t*-test as a hypothesis test. The distribution plots of the accuracies and the permutation test results are shown in Figure 6. The permutation test result indicates that the sdFNC features yield a significantly higher prediction accuracy when compared with tdFNC features, providing evidence that exploiting variability in the spatial domain yields meaningful distinguishing information. The average prediction accuracy using tdFNC features is around 50%, which is equivalent to providing random guesses regarding the class of a subject. This provides additional evidence that tdFNC features are not providing any additional information as compared to a random classifier. The permutation test result between the HC and the SZ group indicates a significantly higher prediction accuracy for the SZ group using sdFNC features. Since the feature used in this technique is the probability of occurrence of each state, we can infer that patients with schizophrenia tend to stay or transition to a certain group of states more often than healthy controls. A natural question is the identification of these predictable states and their differences with respect to states associated to a healthy group of subjects. In the next section we discuss the results obtained from the state-based analysis using the sdFNC matrices and identify the states that are associated with the patients and controls group.

### 3.2. Analysis of States

**Figure 7:**
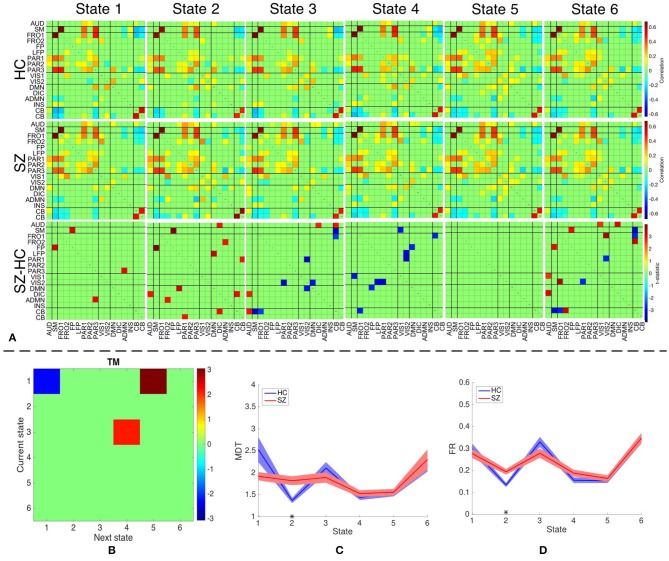
**(A)** The top two rows shows the group-specific states obtained using sdFNC matrices. The bottom row corresponds to the features that demonstrated significant difference (*p* < 0.05, corrected) between HC and SZ group. Red indicates higher value for SZ whereas blue indicates higher value for HC. **(B)** Transition matrix (TM) with each element in the matrix showing transitions that are significantly (*p* < 0.05, corrected) different. Blue indicates HCs transitioned more frequently from current state to next state whereas red indicates SZs transitioned more frequently from current state to next state. **(C)** Mean dwell time of each state for the HC and SZ group. **(D)** Fraction of time spent (FR) in each state by the HC group and SZ group. Results indicate that SZ subjects tend to transition more frequently from State 3 to State 4 whereas those obtained using dsFNC graphs indicate that SZ subjects transition more frequently from State 1 to State 5. We also observe that SZ subjects tend to stay more in State 2.

We identify six distinct states using both temporal and spatial FNC matrices using the method described in section 2.5. The number of clusters is estimated as six using the silhouette criterion. We also compute the optimal number of clusters using other criteria available in the group ICA for fMRI toolbox. The estimated values are in the range 2–10, with the median value being six. Hence, we choose the final values as six for the optimal number of clusters. The group-specific states and features that demonstrate significant differences between HC and SZ group using sdFNC matrices are shown in Figure 7A. The significantly different features within each state were identified by performing a permutation test between the HC group and the SZ group. The group-specific states show differences in the level of connectivity between pairs of components, which are reflected in the third row of Figure 7A that shows differences between the HC and SZ group. The parietal component has high positive connectivity with the auditory, sensorimotor and frontal components in all states and indicates simultaneous activation of these regions. The parietal lobe plays a vital role in processing sensory information such as touch, sound and vision, which is obtained from different parts of the body. A subject in the scanner is exposed to scanner noise and hence the brain is involved in processing the auditory information, causing activation of parietal and auditory components. The parietal component also plays a role in receiving signals from sensory organs, which is then passed to motor-related regions, such as sensorimotor and frontal components, in order to control the body posture. Since a subject is asked to lay still in the scanner, the subject is focusing on balancing his/her body, causing the activation of these regions. An observed positive correlation between the sensorimotor and frontal component provides additional support toward the hypothesis. Cerebellum on the other hand, receives the sensory information from different parts of the body. Hence, a high negative correlation between the parietal and cerebellum component indicates simultaneous deactivation of one component while the other is active, suggesting a process of first receiving and then processing the sensory information. This might also help explain the observed negative correlation between cerebellum and motor-related components. These connections are observed in all states, indicating that these regions form a central hub at resting-state and play a vital role resting-state fMRI data.

We obtain the transition matrix, dwell times and fraction of time spent in each state for each subject as described in section 2.5. For each transition pair {*i, j*}, *i, j* = 1, …, 6, we perform a permutation test to identify differences between the HC and the SZ group. Each significantly different pair denotes that one group transitioned from state *i* to *j* more frequently than the other group. Similarly, we perform a permutation test on the mean dwell time of each state and fraction of time spent in each state to test for differences between HC and SZ group. The results for transition matrices (TM), mean dwell time (MDT) and fraction of time spent (FR) are shown in Figures 7B–D, respectively. The transition matrix indicates that healthy controls tend to stay in State 1 more frequently, whereas patients with schizophrenia tend to transition more frequently from State 3 to State 4 and State 1 to State 5. State 3 and 4 differ in the level of positive correlation between cerebellum and auditory component, insular and parietal component, visual and parietal component and anterior DMN and visual component, whereas State 1 and 5 differ in the level of positive correlation within the visual network, and between the cerebellum and visual component. These states also differ in the level of negative correlation between the cerebellum and left fronto-parietal component. These connections are also observed in State 2 where patients demonstrate a significantly higher mean dwell time and fraction of time spent compared to controls. Hence patients with schizophrenia tend to reside in or switch to a state that has high positive correlation within the visual network and between the anterior DMN and frontal component, visual and parietal component, anterior DMN and frontal component, and cerebellum and visual component. The patients group also tend to reside in or switch to a state that has high negative correlation between the cerebellum and left fronto-parietal component. This suggests that patients with schizophrenia are associated to a hyperconnected brain network and studies have shown their tendency to engage more brain regions than healthy controls (Ma et al., 2011; Ćurčić-Blake et al., 2015; Walther et al., 2017).

Since patients with schizophrenia demonstrate a significantly high mean dwell time and fraction of time spent in State 2, and controls show a high (although not significant) mean dwell time in State 1, we discuss these two states in detail. State 2 differs from State 1 in terms of high positive correlation within the visual network, between frontal and anterior DMN component, cerebellum and parietal component, cerebellum and visual component, and DMN and insular component. A high negative correlation is also observed between the frontal and visual component, parietal and anterior DMN, DMN and anterior DMN. As discussed above, a high negative correlation between parietal and cerebellum component is due to the cognitive process of receiving and processing sensory information one at a time, a positive correlation between these components in State 2 suggests abnormal connectivity. A healthy brain has shown evidence of positive correlation between anterior and posterior DMN, and a deactivation in DMN due to an activated INS region (Sridharan et al., 2008; Nekovarova et al., 2014). However a high negative correlation between the anterior DMN and posterior DMN, and a high positive correlation between posterior DMN and insular region in State 2 of the SZ group also provides evidence of dysfunction in the DMN domain of schizophrenia, which is a common trait in this group (Nekovarova et al., 2014). A high positive correlation between anterior DMN and frontal component might suggest the activation of both region due to their role in social behavior and impulse control. Patients with schizophrenia are known to have paranoia traits, causing them to be constantly aware of the surroundings and prone to impulse control disorder. This causes hyperactivity in the DMN and frontal components of schizophrenic patients (Fusar-Poli et al., 2011; Guo et al., 2017; Zhou et al., 2019). The bottom row of Figure 7 indicates the connections that demonstrated significant difference (*p* < 0.05, corrected) between the HC and SZ group. High absolute connectivity is SZ group is indicated by red while high absolute connectivity in the HC group is indicated by blue. State 2 shows most connections that have significantly high absolute correlation in the SZ group. Patients exhibit high correlation between the cerebellum and parietal component, posterior and anterior DMN component, posterior DMN and left fronto-parietal, auditory and DIC component, and cerebellum and DIC network. A significantly high correlation between these components in the SZ group suggest a hyperconnected DMN, which is a common trait of patients with schizophrenia (Garrity et al., 2007; Whitfield-Gabrieli et al., 2009). A significantly higher connectivity between the anterior DMN and frontal component, and parietal and cerebellum component provides additional support to the hypothesis of paranoia and abnormal behavior in schizophrenia patients.

## 4. Conclusion and Future Work

Dynamic functional connectivity analysis is widely studied in the temporal domain. However there are also substantial dynamics present in the spatial variability across networks, an understudied area. In this work, we explore the benefits of exploiting the variability in the spatial domain using a prediction technique. Our results indicate that for resting-state fMRI data, the use of spatial dFNC matrices provides meaningful distinguishing characteristics from healthy controls and patients with schizophrenia. We also observe a higher prediction accuracy for the patients group compared with healthy controls, indicating that patients are more likely to stay in or switch among a particular group of states. We also identify the states associated to patients with schizophrenia and study the characteristics of these states. Our results indicate that patients with schizophrenia tend to stay in or switch to a state corresponding to a hyperconnected brain network. In additional, sdFNC features show evidence of significant association of spatial networks to a measure of paranoia in schizophrenia group, highlighting the benefit of the proposed approach as a possible biomarker of illness.

The higher predictability of the sdFNC features and its ability to capture discriminating features, enables the analysis of dFNC in the spatial domain, and leads to a number of future directions. A study to compare different sliding window lengths can be applied to identify a range of lengths suitable for capturing dFNC patterns in the spatial domain. Due to large number of samples in this domain, the sliding window length can be reduced below 30 s as well, in order to capture highly fluctuating networks of interest. This study would not have been possible with conventional methods that use time courses to study dFNC patterns, due to limited number of samples. In this study we identified states from sdFNC patterns and obtained state-based metrics such as transition matrix, mean dwell time and fraction of time spent. Other metrics derived from graph-theoretical analysis such as connectivity strength, modularity and centrality can also be obtained. Different robust clustering approaches can be used to obtain states and compared with the method used in this paper. The study of dynamic functional connectivity is prominent during resting-state during which the neuronal activity is under no constraint as compared with task-related fMRI. However, the benefits of spatial dynamics can be explored under task-constraints. The main focus of this paper is to determine the power of the spatial dynamic features and not achieving a high classification accuracy. Hence, we use a simple Naïve Bayes classifier for predicting the subject class, which ensures that the classification rates would be as independent as possible from the tuning of classifier parameters. However, the prediction accuracy can be improved by using complex classifiers such as kernel support vector machines or neural networks, e.g., a seed-based approach obtained a classification accuracy of 86.3% by using a support vector machine classifier (Kottaram et al., 2018).

## Data Availability Statement

Publicly available datasets were analyzed in this study. This data can be found here: https://coins.trendscenter.org/.

## Author Contributions

SB contributed the primary research idea, implementation, experimentation, organization, and primary writing. QL and VC provided valuable insights toward understanding the results and helped with writing and organization. TA directed the research, identified the direction and problems to investigate, provided feedback, and helped with writing.

### Conflict of Interest

The authors declare that the research was conducted in the absence of any commercial or financial relationships that could be construed as a potential conflict of interest.

## References

[B1] Adali T. Anderson M. Fu G.-S. (2014). Diversity in independent component and vector analyses: identifiability, algorithms, and applications in medical imaging. IEEE Sig. Process. Mag. 31, 18–33. 10.1109/MSP.2014.2300511

[B2] Aine C. Bockholt H. Bustillo J. Canive J. Caprihan A. Gasparovic C.. (2017). Multimodal neuroimaging in schizophrenia: description and dissemination. NeuroInformatics 15, 343–364. 10.1007/s12021-017-9338-9 28812221 PMC5671541

[B3] Allen E. A. Damaraju E. Plis S. M. Erhardt E. B. Eichele T. Calhoun V. D. (2014). Tracking whole-brain connectivity dynamics in the resting state. Cereb. Cortex 24, 663–676. 10.1093/cercor/bhs352 23146964 PMC3920766

[B4] Anderson M. Adali T. Li X.-L. (2012). Joint blind source separation with multivariate Gaussian model: algorithms and performance analysis. IEEE Trans. Sig. Process. 60, 1672–1683. 10.1109/TSP.2011.2181836

[B5] Bhinge S. Long Q. Levin-Schwartz Y. Boukouvalas Z. Calhoun V. D. Adali T. (2017). Non-orthogonal constrained independent vector analysis: application to data fusion, in International Conference on Acoustics, Speech and Signal Processing (ICASSP) (New Orleans, LA), 2666–2670.

[B6] Bhinge S. Mowakeaa R. Calhoun V. D. Adali T. (2019). Extraction of time-varying spatio-temporal networks using parameter-tuned constrained IVA. IEEE Trans. Med. Imaging. 38, 1715–1725. 10.1109/TMI.2019.2893651 30676948 PMC7060979

[B7] Boukouvalas Z. Levin-Schwartz Y. Adali T. (2017). Enhancing ICA performance by exploiting sparsity: application to FMRI analysis, in IEEE International Conference on Acoustics, Speech and Signal Processing (ICASSP) (New Orleans, LA: IEEE), 2532–2536.

[B8] Calhoun V. D. Adali T. (2012). Multisubject independent component analysis of fMRI: a decade of intrinsic networks, default mode, and neurodiagnostic discovery. IEEE Rev. Biomed. Eng. 5, 60–73. 10.1109/RBME.2012.2211076 23231989 PMC4433055

[B9] Calhoun V. D. Adali T. Pearlson G. Pekar J. (2001a). Group ICA of functional MRI data: separability, stationarity, and inference, in Proceedings of the International Conference on ICA and BSS San Diego, CA. Vol. 155.

[B10] Calhoun V. D. Adali T. Pearlson G. D. Pekar J. (2001b). A method for making group inferences from functional MRI data using independent component analysis. Hum. Brain Mapp. 14, 140–151. 10.1002/hbm.1048 11559959 PMC6871952

[B11] Calhoun V. D. de Lacy N. (2017). Ten key observations on the analysis of resting-state functional MR imaging data using independent component analysis. NeuroImaging Clin. 27, 561–579. 10.1016/j.nic.2017.06.012 28985929 PMC5657522

[B12] Calhoun V. D. Kiehl K. A. Pearlson G. D. (2008). Modulation of temporally coherent brain networks estimated using ICA at rest and during cognitive tasks. Hum. Brain Mapp. 29, 828–838. 10.1002/hbm.20581 18438867 PMC2649823

[B13] Chang C. Glover G. H. (2010). Time–frequency dynamics of resting-state brain connectivity measured with fMRI. Neuroimage 50, 81–98. 10.1016/j.neuroimage.2009.12.011 20006716 PMC2827259

[B14] Cribben I. Haraldsdottir R. Atlas L. Y. Wager T. D. Lindquist M. A. (2012). Dynamic connectivity regression: determining state-related changes in brain connectivity. NeuroImage 61, 907–920. 10.1016/j.neuroimage.2012.03.070 22484408 PMC4074207

[B15] Ćurčić-Blake B. van der Meer L. Pijnenborg G. H. David A. S. Aleman A. (2015). Insight and psychosis: functional and anatomical brain connectivity and self-reflection in Schizophrenia. Hum. Brain Mapp. 36, 4859–4868. 10.1002/hbm.22955 26467308 PMC6869637

[B16] Du W. Ma S. Fu G.-S. Calhoun V. D. Adali T. (2014). A novel approach for assessing reliability of ICA for fMRI analysis, in International Conference on Acoustics, Speech and Signal Processing (ICASSP) (Florence: IEEE), 2084–2088.

[B17] Fox M. D. Snyder A. Z. Vincent J. L. Corbetta M. Van Essen D. C. Raichle M. E. (2005). The human brain is intrinsically organized into dynamic, anticorrelated functional networks. Proc. Natl. Acad. Sci. U.S.A. 102, 9673–9678. 10.1073/pnas.0504136102 15976020 PMC1157105

[B18] Fusar-Poli P. Howes O. Allen P. Broome M. Valli I. Asselin M.. (2011). Abnormal prefrontal activation directly related to pre-synaptic striatal dopamine dysfunction in people at clinical high risk for psychosis. Mol. Psychiatry 16:67. 10.1038/mp.2009.108 19949389

[B19] Garrity A. G. Pearlson G. D. McKiernan K. Lloyd D. Kiehl K. A. Calhoun V. D. (2007). Aberrant “default mode” functional connectivity in schizophrenia. Amer. J. Psychiatry 164, 450–457. 10.1176/ajp.2007.164.3.450 17329470

[B20] Guo W. Liu F. Chen J. Wu R. Li L. Zhang Z.. (2017). Hyperactivity of the default-mode network in first-episode, drug-naive schizophrenia at rest revealed by family-based case–control and traditional case–control designs. Medicine 96:e6223. 10.1097/MD.0000000000006223 28353559 PMC5380243

[B21] Hero A. O. Rajaratnam B. (2016). Foundational principles for large-scale inference: illustrations through correlation mining. Proc. IEEE 104, 93–110. 10.1109/JPROC.2015.2494178 27087700 PMC4827453

[B22] Hutchison R. M. Womelsdorf T. Allen E. A. Bandettini P. A. Calhoun V. D. Corbetta M.. (2013). Dynamic functional connectivity: promise, issues, and interpretations. NeuroImage 80, 360–378. 10.1016/j.neuroimage.2013.05.079 23707587 PMC3807588

[B23] Keilholz S. D. Magnuson M. E. Pan W.-J. Willis M. Thompson G. J. (2013). Dynamic properties of functional connectivity in the rodent. Brain Connect. 3, 31–40. 10.1089/brain.2012.0115 23106103 PMC3621313

[B24] Kottaram A. Johnston L. Ganella E. Pantelis C. Kotagiri R. Zalesky A. (2018). Spatio-temporal dynamics of resting-state brain networks improve single-subject prediction of schizophrenia diagnosis. Hum. Brain Mapp. 39, 3663–3681. 10.1002/hbm.24202 29749660 PMC6866493

[B25] Kunert-Graf J. M. Eschenburg K. M. Galas D. J. Kutz J. N. Rane S. D. Brunton B. W. (2018). Extracting reproducible time-resolved resting state networks using dynamic mode decomposition. bioRxiv 343061. 10.1101/343061 PMC6834549 31736734

[B26] Lee M. H. Smyser C. D. Shimony J. S. (2013). Resting-state fMRI: a review of methods and clinical applications. Amer. J. Neuroradiol. 34, 1866–1872. 10.3174/ajnr.A3263 22936095 PMC4035703

[B27] Leonardi N. Van De Ville D. (2015). On spurious and real fluctuations of dynamic functional connectivity during rest. Neuroimage 104, 430–436. 10.1016/j.neuroimage.2014.09.007 25234118

[B28] Li X. Zhu D. Jiang X. Jin C. Zhang X. Guo L.. (2014). Dynamic functional connectomics signatures for characterization and differentiation of PTSD patients. Hum. Brain Mapp. 35, 1761–1778. 10.1002/hbm.22290 23671011 PMC3928235

[B29] Li X.-L. Adali T. (2010). Blind spatiotemporal separation of second and/or higher-order correlated sources by entropy rate minimization, in International Conference on Acoustics Speech and Signal Processing (ICASSP) (Dallas, TX: IEEE), 1934–1937.

[B30] Li Y.-O. Adali T. Calhoun V. D. (2007). Estimating the number of independent components for functional magnetic resonance imaging data. Hum. Brain Mapp. 28, 1251–1266. 10.1002/hbm.20359 17274023 PMC6871474

[B31] Liégeois R. Ziegler E. Phillips C. Geurts P. Gómez F. Bahri M. A.. (2016). Cerebral functional connectivity periodically (de)synchronizes with anatomical constraints. Brain Struct. Funct. 221, 2985–2997. 10.1007/s00429-015-1083-y 26197763

[B32] Long Q. Bhinge S. Levin-Schwartz Y. Boukouvalas Z. Calhoun V. D. Adali T. (2018a). The role of diversity in data-driven analysis of multi-subject fMRI data: comparison of approaches based on independence and sparsity using global performance metrics. Hum. Brain Mapp. 40, 489–504. 10.1002/hbm.24389 30240499 PMC6392437

[B33] Long Q. Jia C. Boukouvalas Z. Gabrielson B. Emge D. Adali T. (2018b). Consistent run selection for independent component analysis: application to fMRI analysis, in International Conference on Acoustics, Speech and Signal Processing (ICASSP) (Calgary, AB: IEEE), 2581–2585. 10.1109/ICASSP.2018.8461646

[B34] Ma S. Calhoun V. D. Phlypo R. Adali T. (2014). Dynamic changes of spatial functional network connectivity in healthy individuals and schizophrenia patients using independent vector analysis. NeuroImage 90, 196–206. 10.1016/j.neuroimage.2013.12.063 24418507 PMC5061129

[B35] Ma S. Eichele T. Correa N. M. Calhoun V. D. Adali T. (2011). Hierarchical and graphical analysis of fMRI network connectivity in healthy and schizophrenic groups, in IEEE International Symposium on Biomedical Imaging: From Nano to Macro (Chicago, IL: IEEE), 1031–1034. 10.1109/ISBI.2011.5872577

[B36] Nekovarova T. Fajnerova I. Horacek J. Spaniel F. (2014). Bridging disparate symptoms of schizophrenia: a triple network dysfunction theory. Front. Behav. Neurosci. 8:171. 10.3389/fnbeh.2014.00171 24910597 PMC4038855

[B37] Preti M. G. Bolton T. A. Van De Ville D. (2017). The dynamic functional connectome: state-of-the-art and perspectives. NeuroImage 160, 41–54. 10.1016/j.neuroimage.2016.12.061 28034766

[B38] Sakoğlu Ü. Pearlson G. D. Kiehl K. A. Wang Y. M. Michael A. M. Calhoun V. D. (2010). A method for evaluating dynamic functional network connectivity and task-modulation: application to schizophrenia. Magn. Reson. Mater. Phys. Biol. Med. 23, 351–366. 10.1007/s10334-010-0197-8 20162320 PMC2891285

[B39] Scott A. Courtney W. Wood D. De la Garza R. Lane S. Wang R.. (2011). COINS: an innovative informatics and neuroimaging tool suite built for large heterogeneous datasets. Front. Neuroinformatics 5:33. 10.3389/fninf.2011.00033 22275896 PMC3250631

[B40] Sridharan D. Levitin D. J. Menon V. (2008). A critical role for the right fronto-insular cortex in switching between central-executive and default-mode networks. Proc. Natl. Acad. Sci. U.S.A. 105, 12569–12574. 10.1073/pnas.0800005105 18723676 PMC2527952

[B41] Varoquaux G. Gramfort A. Pedregosa F. Michel V. Thirion B. (2011). Multi-subject dictionary learning to segment an atlas of brain spontaneous activity, in Biennial International Conference on Information Processing in Medical Imaging (Berlin; Heidelberg: Springer), 562–573. 10.1007/978-3-642-22092-0_46 21761686

[B42] Walther S. Stegmayer K. Federspiel A. Bohlhalter S. Wiest R. Viher P. V. (2017). Aberrant hyperconnectivity in the motor system at rest is linked to motor abnormalities in schizophrenia spectrum disorders. Schizophr. Bull. 43, 982–992. 10.1093/schbul/sbx091 28911049 PMC5581901

[B43] Whitfield-Gabrieli S. Thermenos H. W. Milanovic S. Tsuang M. T. Faraone S. V. McCarley R. W.. (2009). Hyperactivity and hyperconnectivity of the default network in schizophrenia and in first-degree relatives of persons with schizophrenia. Proc. Natl. Acad. Sci. U.S.A. 106, 1279–1284. 10.1073/pnas.0809141106 19164577 PMC2633557

[B44] Yang Z. Craddock R. C. Margulies D. S. Yan C.-G. Milham M. P. (2014). Common intrinsic connectivity states among posteromedial cortex subdivisions: insights from analysis of temporal dynamics. NeuroImage 93, 124–137. 10.1016/j.neuroimage.2014.02.014 24560717 PMC4010223

[B45] Zhou C. Yu M. Tang X. Wang X. Zhang X. Zhang X.. (2019). Convergent and divergent altered patterns of default mode network in deficit and non-deficit schizophrenia. Prog. Neuropsychopharmacol. Biol. Psychiatry 89, 427–434. 10.1016/j.pnpbp.2018.10.012 30367960

